# The Effects of Perceived Neighborhood Immigrant Population Size on Preferences for Redistribution in New York City: A Pilot Study

**DOI:** 10.3389/fsoc.2019.00018

**Published:** 2019-03-26

**Authors:** Liza G. Steele, Krystal M. Perkins

**Affiliations:** ^1^John Jay College of Criminal Justice, City University of New York, New York, NY, United States; ^2^SUNY Purchase, Purchase, New York, NY, United States

**Keywords:** preferences for redistribution, social policy preferences, neighborhood diversity, migration, innumeracy

## Abstract

An extensive literature exists hypothesizing a negative association between immigration and a multitude of social goods issues. Recent analyses, however, have established that the perception of the size of the immigrant population may be more relevant than the actual size of the population in shaping attitudes, and that the effect of immigration on social policy attitudes may be more salient at the local—or even neighborhood—level than at the country-level. In extending this work, we examine how perceptions and misperceptions about the size of the immigrant population affect attitudes about redistribution and social policies within one of the most diverse and ethnically heterogeneous immigrant cities in the world, New York City. We analyzed data from a diverse sample of 320 NYC residents recruited through Amazon Mechanical Turk who responded to a series of questions regarding their perceptions of the size of the immigrant population of their neighborhood before indicating their redistributive and social policy preferences. We found that about a quarter of New Yorkers overestimated the size of the non-citizen population, though the proportion was lower than those in studies of other geographic units. In addition, those that perceived a lower citizen proportion or overestimated the size of the non-citizen population were the least supportive of redistribution and social policies. Implications for the existing research on the relationship between immigration and social policy preferences are discussed.

## Introduction

The transnational movement of people has recently become a highly salient and contested issue in social and political life. In particular, there has been growing tension concerning the hosting of immigrants, which some argue fueled the popularity of right-wing populist parties in Europe and the United States, as well as general exclusionary reactions toward immigrants. Across these distinct national settings, there has been considerable debate regarding citizenship, constructions of national identity, and multicultural diversity with significant ramifications for everyday experience. A key dimension in these immigrant-relevant debates concerns the overall size of the immigrant population. Indeed, a small cohort of studies suggests that variations in exposure to and perceptions about the size of immigrants and immigration have varying implications for social policy attitudes and related phenomena (Senik et al., 2009; Burgoon et al., 2012; Alesina et al., 2018).

In this paper, we consider the question of how underestimations and overestimations of the size of an immigrant population affect attitudes about redistribution and social policy within the immigrant-rich city of New York City (NYC). We believe our research makes a number of important contributions to the immigration literature. First, we focus on NYC residents' subjective perceptions regarding the size of the immigrant population because objective measures have been shown to have limited direct relevance to welfare state attitudes and attitudes about immigrants, more broadly (Semyonov et al., 2004; Spies and Schmidt-Catran, 2016; Alesina et al., 2018; Gorodzeisky and Semyonov, 2019). Second, we ask respondents about their perceptions regarding the size of both the immigrant (specifically, non-citizens) and citizen populations in research participants' respective neighborhoods, and examine the accuracy of these perceptions by comparing their responses to data from the American Community Survey (United States Census Bureau, 2017). Unlike previous studies that have asked respondents about their perceptions and judgments regarding the size of the immigrant population only, our study will allow us to theoretically test and examine directly whether perceptions of the size of the immigrant and citizen populations vary, as well as the extent to which these perceptions are related to preferences for redistribution and social policies.

Third, while many existing studies have focused on locales experiencing immigration as a new phenomenon, our data come from residents living in NYC, a city with a long history of being the home to people of many different immigrant backgrounds and one of the top destinations for international migrants. Finally, to our knowledge, our research is one of the few studies to examine individuals' perceptions and misperceptions about the size of an immigrant population within much smaller geographic units (e.g., neighborhoods) and how such perceptions are related to attitudes about redistribution and social policies. National or cross-national measures regarding the size of an immigrant population are not ideal indicators as they may be too tangential to people's actual contact with and exposure to immigrants. Indeed, some evidence suggests that smaller geographic units, such as neighborhoods of residence, better capture people's everyday experiences (Dinesen and Sønderskov, 2015; Koopmans and Schaeffer, 2016). Together, we believe our paper adds a number of important dimensions to the literature on immigrant group size and policy preferences. Before presenting the specifics of our study, the next section provides a brief overview of previous research that examines the links between perceptions of proportions of citizens and non-citizens—primarily at the country level—the accuracy of those perceptions, and policy preferences.

## Immigration, Public Spending, and Preferences for Redistribution: Existing Evidence

In Alesina and Glaeser's (2004) seminal book, the authors contend that large-scale immigration will weaken the welfare state in Europe. Using macro-level indicators across 54 countries, they established a negative association between “racial fractionalization” and social welfare spending. In particular, they find that highly homogenous countries in Europe, like the Nordic countries, had very generous welfare systems, while, highly heterogeneous countries, such as many Latin American countries, had weak welfare states. Following Alesina and Glaeser (2004), a number of studies have critically examined immigration's potential consequences for the welfare state in different contexts using different indicators including fiscal burden, public spending, and attitudes about redistribution (Soroka et al., 2006; van Oorschot, 2006; Brady and Finnigan, 2014). In a study by Soroka et al. (2006), for example, the authors find that across three decades, social spending grew less in nations with higher rates of immigration than in countries with lower immigration rates. Other work has similarly concluded that immigration and ethnic heterogeneity, more broadly, are strong negative predictors of social welfare spending (Sanderson, 2004; Sanderson and Vanhanen, 2004; Vanhanen, 2004). A related set of scholarship has also investigated the association between attitudes about redistribution and the presence of immigrants across various countries and within different states in the United States (Mau and Burkhardt, 2009; Eger, 2010; Brady and Finnigan, 2014; Steele, 2016). Comparing 17 European countries, Mau and Burkhardt (2009) find that countries with higher percentages of a non-Western foreign-born cohort tend to be less supportive of government redistribution. Within the context of the United States, studies have also documented significant negative effects related to the prevalence of immigrants on states' welfare programs (Fox, 2004; Fox et al., 2013).

Other scholarship, however, has reported little to no association between welfare spending or attitudes about redistribution and the presence of and size of immigrant populations (Senik et al., 2009; Hainmueller and Hiscox, 2010). Some empirical work suggests that it is not actual immigration that influences welfare spending and redistribution attitudes, but *how* immigration is perceived and experienced. In line with these ideas, previous research has found that respondents in professions with large portions of immigrants were more supportive of redistribution than respondents in occupations with low shares of immigrants (Burgoon et al., 2012). In contrast, nationally, a high foreign-born population was unrelated to support for redistribution. Similarly, Senik et al.'s (2009) large-scale analysis of 22 European countries found a weak association between immigration and endorsement of government redistribution. They do find, however, that support for the welfare state was weakest among those who were averse to immigrants and express apprehension about the fiscal implications of immigration.

Scholars have also begun to consider the question of how people's perceptions of the number of immigrants are related to redistribution attitudes. These studies draw from a related set of studies that have demonstrated that perceptions of the size of the immigrant population are often distorted and these misperceptions, in turn, are associated with anti-immigrant attitudes (Semyonov et al., 2004, 2006; Herda, 2013; Pottie-Sherman and Wilkes, 2017). Gorodzeisky and Semyonov (2019), for example, find that the more respondents misperceive the size of the immigrant population, the greater their anti-immigrant sentiments. In extending these ideas to attitudes about government redistribution, Alesina et al. (2018) present the results of a large-scale study across six countries (France, Germany, Italy, Sweden, the U.K., and the United States). One of the major contributions of this work and relevant to the present paper was their finding regarding the degree of misperceptions about the number and composition of immigrants and its relationship to redistribution attitudes. Across all countries studied, respondents overestimated the number of immigrants, particularly Muslim immigrants and immigrants from Middle Eastern and North African countries. These misperceptions, in turn, were associated with more negative attitudes toward redistribution.

In summary, there appears to be a distinct set of contextual and individual-level characteristics related to immigration (rising immigration, misperceptions of the size of the immigrant group) and attitudes about redistribution and related phenomena. As noted earlier, there is growing evidence to suggest that the perception of the size of the immigrant population may be more relevant than the actual size of the population in shaping attitudes (Alesina et al., 2018; Gorodzeisky and Semyonov, 2019), and that the effect of immigration on social policies may be more salient at more local or personal levels, than at the national or cross-national level (Fox, 2004; Burgoon et al., 2012; Fox et al., 2013). We evaluate these possibilities in the case of NYC, using data collected via an online survey. We focus on NYC residents' subjective perceptions regarding the size of the immigrant (specifically, non-citizen) and citizen population, and examine the accuracy of these perceptions by comparing their responses to data from the American Community Survey. NYC is a distinct context in which to explore these possibilities because of the city's multilayered history of immigration. According to recent Census data, 40 percent of the NYC population was born outside of the United States. More than 150 countries comprise NYC's immigrant population and immigrants from the Dominican Republic and China are the largest foreign-born groups. The borough of Queens is the most immigrant-dense borough and Elmhurst in Queens has the highest share of immigrants with nearly seventy-five percent of its residents foreign-born. At the same time however, NYC is also very highly segregated residentially with immigrants typically living in ethnic minority or immigrant-dense neighborhoods and U.S. citizens, particularly white U.S. citizens, living in neighborhoods with high proportions of white Americans.

In the present study, we examine specific expectations of perceptions and misperceptions on attitudes about redistribution and social policy. We were first interested in how NYC respondents perceive the size of the immigrant population in their respective neighborhoods. On the basis of studies that have demonstrated that people's perceptions of immigrant populations are often distorted, we hypothesized that people will be more likely to overestimate the size of the immigrant population in their respective neighborhood than to underestimate or accurately estimate (H1). However, it is also possible that our respondents will be less likely than those in other contexts to overestimate the proportion of the immigrant population. Gorodzeisky and Semyonov (2019) suggest that native-born citizens in countries with higher percentages of immigrants, as compared to citizens of “new immigration” countries, have longer experience with migration and may have had more opportunities to develop accurate knowledge regarding the actual size of the immigrant population. In line with these ideas, because New York City has the largest foreign-born population among cities in the United States (and second-largest globally after London) and immigrants have been so entrenched into the fabric of NYC life, NYC residents might perceive their presence as unproblematic and have more defined knowledge regarding their overall size. Therefore, we propose an alternative hypothesis: NYC residents will be less likely (than those in studies of other geographic areas) to overestimate the size of their neighborhood's immigrant population (H2).

We were also interested in whether, and in what way, perceptions and accuracy of perceptions (misperceptions) are associated with redistribution attitudes. Adding to the growing body of literature examining perceptions and misperceptions of the size of the immigrant population and attitudes about redistribution, we hypothesized that those who perceive (regardless of their accuracy) higher proportions of immigrants will be less supportive of redistribution and social policies in comparison to those who perceive lower proportions (H3). Moreover, overestimated perceptions will be associated with lower support for redistribution and social policies (H4).

## Data and Methods

We conducted an original online survey in May 2015 that asked participants a range of questions about their neighborhoods, and assessed their preferences for redistribution and specific social policies. We obtained informed consent from all participants prior to their participation in the online survey. The study was conducted in accordance with the protocol approved by the Institutional Review Board of the State University of New York, Purchase College (IRB Protocol Number 141561). We recruited and paid participants (*N* = 346) via Amazon Mechanical Turk (mTurk), and required that they live in New York City and have a good performance rating on mTurk^1^. The sample was 39.2% female, 60.8% white, and 93.3% native-born citizen with a median age of 30. In Table 1, we present a comparison of the demographics of our sample with those of the populations of New York City and the U.S. Typical of mTurk samples^2^, the demographics of our sample differ from the general population of both the U.S. and New York City. Despite such differences, a growing body of research indicates that mTurk is a valid tool for the study of political attitudes (Clifford et al., 2015; Thibodeau and Flusberg, 2017).

**Table 1:** Demographics of mTurk sample vs. NYC and U.S. populations.

**Variable**	**Sample median or %**	**NYC median or %^**a**^**	**U.S. median or %^**b**^**
Female	39.2%	52.3%	50.8%
Age	30.0	35.9	37.8
**RACE/ETHNICITY**^**c**^			
White (not Hispanic/Latinx)	60.8%	32.3%	61.5%
Black	13.7%	22.2%	13.1%
Latinx	11.0%	29.1%	17.6%
Asian (East or South)	13.7%	13.6%	5.5%
Other	0.8%	1.0%	5.9%
Multiracial	7.4%	1.8%	3.1%
**CITIZENSHIP STATUS**			
U.S. citizen (U.S. born)	93.3%	62.8%	86.5%
U.S. citizen (born abroad)	5.1%	20.2%	6.4%
Legal permanent resident	1.6%		
**ACS CATEGORIES**			
Not a U.S. citizen	1.6%	17.0%	7.0%
Foreign born	6.7%	37.2%	13.5%
**NYC BOROUGH OF RESIDENCE**			
Bronx	10.6%	17.0%	
Brooklyn	34.8%	30.8%	
Manhattan	23.4%	19.3%	
Queens	25.8%	27.3%	
Staten Island	5.5%	5.6%	
N	256	8,461,989	321,418,821

a *Source: NYC Planning Population FactFinder using 2012–2016 American Community Survey data (United States Census Bureau 2017) (note: these data include those under age 18, who are excluded from our study)*.

b *Source: 2015 American Community Survey data (United States Census Bureau 2017) (note: these data include those under age 18, who are excluded from our study)*.

c *Mutually exclusive race (categories exclude those who selected more than one race, who are included in the “multiracial” category)*.

Data were not analyzed from participants who did not complete the study, did not pass the screening questions, provided problematic information about their neighborhood of residence^3^, or submitted an incorrect completion code. After these exclusion criteria were applied, data from 256 participants remained for analysis. However, because foreign-born respondents are particularly underrepresented in our sample (6.7 vs. 37.2% in the actual NYC population), we limit our analytic sample to native-born respondents. In addition, to facilitate cross-model comparisons, missing data are handled through list-wise deletion yielding a final analytic sample of 201.

## Outcome Measures

We modeled our policy preferences questions on items from the 2009 and 2016 modules of the International Social Survey Programme (ISSP) (ISSP Research Group, 2017, 2018)^4^. The questions from the 2009 wave (“Social Inequality IV”) asked respondents to what extent they agreed or disagreed—where [1] represented “strongly agree,” [2] represented “agree,” [3] represented “neither agree nor disagree,” [4] represented “disagree,” and [5] represented “strongly disagree”—with the following statements: “Differences in income in our country are too large” (“income differences”); “It is the responsibility of the government to reduce the differences in income between people with high incomes and those with low incomes” (“income equality”); “The government should provide a decent standard of living for the unemployed” (“unemployment”); and “The government should spend less on benefits for the poor” (“benefits poor”). In the last question, we changed “less” to “more.” To facilitate a more straightforward interpretation, we reversed the ISSP coding of these items so that [1] represents “strongly disagree” and [5] represents “strongly agree.”^5^ Factor analyses pointed to a three-item index of the income equality, unemployment, and benefits poor items (“Index 1: Redistribution”; Cronbach's alpha = 0.85).

In the 2016 (“Role of Government V”) module of the ISSP, respondents were asked the following about several social policies: “On the whole, do you think it should or should not be the government's responsibility to…” Ordinal answer categories included “definitely should be,” “probably should be,” “probably should not be,” and “definitely should not be.” We included in our study five of the most relevant items from the 2016 questions that were unique from the 2009 questions: “provide a job for everyone who wants one” (“jobs”); “provide health care for the sick” (“health”); “provide a decent standard of living for the old” (“old age”); “give financial help to university students from low-income families” (“student aid”); and “provide decent housing for those who can't afford it” (“housing”). We reversed the original coding of these items so that [1] represents “definitely should not be” and [4] represents “definitely should be.” We constructed an index of the health, old age, student aid, and housing items based on the implications of factor analyses (“Index 2: Social Policies”; Cronbach's alpha = 0.89).

In Table 2, we present mean responses to the questions used to construct the indices; the means of the indices for the analytic sample are presented in Table 3. We also include mean policy preference responses from the ISSP U.S. data, along with a subsample of respondents from large mid-Atlantic cities (specific cities are not identified in the publicly available ISSP data), for comparison.

**Table 2:** Mean support for redistribution and social policies.

**Variable**	**Sample mean (S. D.)**	**U. S. mean (S. D.)**	**Large city mid-atlantic U. S. mean (S. D.)**	**Range**
**INDEX 1: REDISTRIBUTION**^**a**^				
Income equality	3.55 (1.23)	2.66 (1.26)	3.05 (1.26)	[1, 5]
Unemployment	3.71 (1.10)	3.10 (1.18)	3.63 (1.00)	[1, 5]
Benefits for the poor	3.53 (1.21)	3.53 (1.04)	3.59 (1.10)	[1, 5]
Number of observations	320	1,405	91	
**INDEX 2: SOCIAL POLICIES**^b^				
Health	3.11 (0.99)	3.33 (0.79)	3.71 (0.56)	[1, 4]
Old age	3.12 (0.94)	3.34 (0.74)	3.54 (0.55)	[1, 4]
Student aid	3.00 (0.95)	3.32 (0.75)	3.70 (0.56)	[1, 4]
Housing	2.87 (0.94)	2.99 (0.81)	3.38 (0.74)	[1, 4]
Number of observations	320	1,315	40	

a *U.S. national and regional data from 2009 ISSP (ISSP Research Group 2017); ISSP-provided analytic weights applied*.

b *U.S. national and regional data from 2016 ISSP (ISSP Research Group 2018); ISSP-provided analytic weights applied*.

**Table 3:** Summary statistics, analytic sample.

**Variable**	**Sample mean, median category, or %**	**SD**	**Range**
**OUTCOME MEASURES**			
Index 1: redistribution	3.58	1.09	1, 5
Index 2: social policy	3.07	0.83	1, 4
**CONTROL MEASURES**			
Female	40.3%		
Age	31.7	9.5	19, 63
**RACE/ETHNICITY**			
White (not Hispanic/Latinx)	64.2%		
Black	12.4%		
Latinx	11.0%		
Asian (East or South)	11.5%		
Other	1.0%		
College completed	65.7%		
Income	$50,001–$75,000		$0, > $1 million
Employed in high-immigration sector^a^	42.9%		
**PERCEPTION: # OF WHITE NEIGHBORS**			
Low	18.4%		
Medium	44.8%		
High	36.8%		
N	201		

a *n = 177 for this measure only*.

## Key Explanatory Measures

Respondents' neighborhoods of residence were determined via a write-in question (“What is the name of the NYC neighborhood or community where you currently live?”) and their zip codes.

To measure perceptions of the size of the neighborhood immigrant population, we asked respondents to estimate the proportion of citizens, documented immigrants, and undocumented immigrants in their neighborhoods. In our study, the questions about perceived proportion of immigrants in a community are modeled on similar items from the Project on Race and Ethnicity in Latin America (PERLA) surveys (Telles, 2013). We asked respondents to estimate the proportion of their neighbors who were American citizens, documented immigrants (residents with green cards and other types of work visas), and undocumented immigrants using the following prompt: “In the present, in the neighborhood or community where you live, how many of your neighbors are …” (possible responses included “none,” “almost none,” “very few,” “less than half,” “close to half,” and “more than half”). We opted for ordinal answer categories over asking respondents to guess the exact number precisely because previous studies had already shown that such estimations were highly error-prone, with majority- and minority-group members in both the U. S. and Europe being very likely to overestimate the size of minority or immigrant populations (Nadeau et al., 1993; Sigelman and Niemi, 2001; Herda, 2010). For example, one of the earliest studies on this topic, known as population “innumeracy,” showed that Americans thought three-quarters of the country's population were black, Hispanic, or Jewish (Nadeau et al., 1993). Notably, missingness in the analytic sample is primarily attributable to non-response on the perceptions of non-citizens questions (7% non-response for the documented immigrant question and 12.5% for the undocumented immigrant question), which is much higher than for the perceptions of citizens' question (3% non-response).

To ensure a large enough number of observations per category of analysis in the presentation of some results, perception measures are recoded into three-categories (“3-category”): “low” (none, almost none, very few), “medium” (less than half, close to half), and “high” (more than half) levels of a group.

To compare our respondents' perceptions to the actual proportions, we geocoded each neighborhood to match the city's community districts, for which detailed population information is available via the American Community Survey (ACS), which is conducted annually (United States Census Bureau, 2017). We generate variables representing the percentage of a community district that is native-born (which includes being born in Puerto Rico and U.S. island areas, along with being born abroad to American parents; ranging from 37 to 85%) and non-citizen (ranging from 4 to 36%); in some parts of the paper, we refer to other variables measuring the foreign-born (ranging from 15 to 63%), naturalized citizen (ranging from 8 to 38%), and recent immigrant populations (foreign-born entering the U.S. in the year 2000 or later; ranging from 3 to 29%). As an example to illustrate the meanings of these measures, 60 percent of the Jackson Heights/North Corona neighborhood of Queens is foreign-born, 26 percent of residents immigrated in the year 2000 or later, and 36 percent of residents are not citizens. On the other end of the spectrum, 15 percent of the population of the Tottenville/Great Kills/Annadale neighborhood in Staten Island is foreign-born, with 3 percent of residents being more recent immigrants and 3.5% being non-citizens. Notably, even the NYC neighborhood with the lowest proportion of foreign-born residents (Tottenville/Great Kills/Annadale) is still above the national rate (13% in 2015). Because our neighborhood questions specifically measured perceptions of the proportion of documented and undocumented immigrants, the non-citizen figures from the ACS data are the most pertinent to our analyses. As illustrated by the examples above, unsurprisingly but notably, proportions of foreign-born and non-citizens by neighborhood follow similar patterns.

Following Herda (2013) and Gorodzeisky and Semyonov (2019), we use three distinct qualitative categories of the accuracy of perceptions of the size of the citizen and non-citizen populations: accurate estimation, overestimation, and underestimation. We compared estimates of the neighborhood citizen population to ACS data on neighborhood native-born population. We also compared estimates of the perceptions of documented and undocumented neighborhood residents to the ACS data on non-citizen population by neighborhood. We then generated a categorical variable in which [1] represents accurate estimation, [2] represents under-estimation, and [3] represents over-estimation. For details about the classification of respondents into these categories, please see Table A1 in Supplementary Material.

## Control Variables

Because citizenship and whiteness may be conflated in the American context, in some models we control for the perceived size of the white population. Previous research has demonstrated that both white and minority respondents who perceive a larger white population in their NYC neighborhoods were less supportive of redistribution and social policies (Steele and Perkins, 2018). Other recent research has shown that assumptions about immigration status are related to national origin (Flores and Schachter, 2018), often a proxy for whiteness in the U.S. context. Using the same prompt and answer categories described for the perception of citizens question, we asked respondents to estimate the proportion of white people in their neighborhoods. We include this measure to control for any possible conflation of whiteness and citizenship.

We also expect that the effects of support for redistribution and social policy will vary by the race/ethnicity of respondents themselves. We use a standard measure of race/ethnicity, asking “What is your race/ethnicity? (Please choose all that apply).” The racial and ethnic composition of our sample is consistent with the composition of the U.S. population, although it is not fully reflective of the diversity of New York City. Moreover, unlike many mTurk studies, our data include a substantial proportion of responses from black (14%), Latinx (11%), and Asian (14%) respondents.^6^ However, for the sake of parsimony, we simply control for white vs. minority status.

In some cases, we also control for demographic characteristics including gender, age, level of education (1 = college completed), and household income. In addition, following Alesina et al. (2018), we construct a dummy variable for respondents who work in high-immigration sectors, defined as sectors in which the share of immigrants working in that sector is higher than the average share of immigrants employed in the country. Because of missing data, we only include the high-immigrant sector measure in tests of robustness. Summary statistics for the control measures are presented in Table 3.

## Analytical Strategy

We conduct a preliminary analysis of the relationship between perceived neighborhood immigrant population size, the accuracy of those perceptions, and support for redistribution and social policy using the results of our mTurk pilot study. Given that the outcome variables are additive indices with 13 unique values each, we treat them as continuous and model ordinary least squares (OLS) regressions^7^. As a test of robustness, which is included in the Appendix in Supplementary Material, we also estimate OLS regression models with robust, clustered standard errors, which account for the presence of unobserved, neighborhood-level dependence in the error terms and adjust for the lack of independence of observations within neighborhoods (Chen et al., 2003; Wooldridge, 2003).^8^

## Results

Below, we begin by examining descriptive findings from our data. Then we analyze the relationship between perceptions about the size of the neighborhood immigrant population and preferences for redistribution and social policy. Finally, we examine the relationship between the effects of the *accuracy* of perceptions of the size of the immigrant population and policy preferences.

## Descriptive Findings

Despite the fact that Amazon Mechanical Turk respondents are typically more liberal than respondents in nationally representative samples (Berinsky et al., 2012; Huff and Tingley, 2015), mTurk samples have been established as valid tools for the evaluation of political attitudes (Clifford et al., 2015; Thibodeau and Flusberg, 2017). Comparing mean responses to redistribution and social policy questions in our study to mean scores from the U.S. samples and the sub-samples of residents of large cities in the mid-Atlantic in the 2009 and 2016 waves of the ISSP (Table 2), we find that the mean responses to the question about support for benefits for the poor were very similar to the results from the ISSP samples. Our respondents appear to be more liberal than their counterparts in terms of attitudes about income equality and unemployment. However, in terms of mean support for health, old age, student aid, and housing policies, our respondents may be more conservative than either the urban mid-Atlantic sample or even the U.S. sample.

If we examine the correlation between neighborhood mean support for redistribution and social policy and the ACS percentages of native-born and non-citizen residents, we find very weak evidence of linear relationships. The Pearson's correlation coefficient for the percentage native-born residents in a neighborhood is −0.18 (statistically significant) and 0.09 (non-significant) for the non-citizen percentage for both attitudinal indices. Thus, we turn to the role of perceptions of the size of these populations.

**Figure 1:**
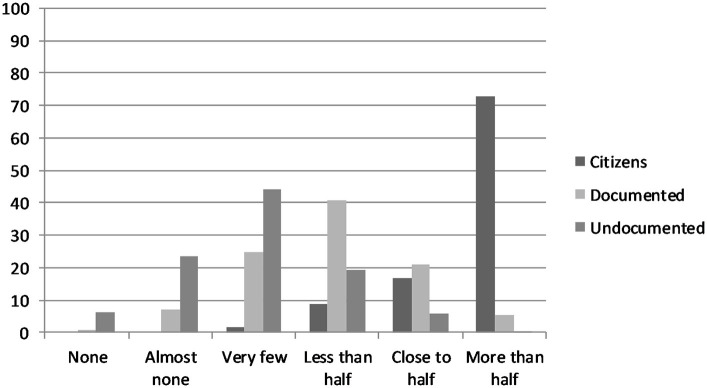
Perceptions of the size of the citizen and immigrant populations in respondent's neighborhood.

The distributions of responses to the questions measuring perceptions of the size of the citizen and non-citizen population are shown in Figure 1. Regarding the proportion of citizens, close to three-quarters of respondents perceived them as the majority in their neighborhoods. Most respondents perceived documented immigrants to have a substantial, but not majority, presence in their neighborhoods. The modal response to the question about the size of the undocumented immigrant population in a respondent's neighborhood was “very few.”

The next stage of our analysis entailed assessing the accuracy of these perceptions. Based on previous studies, we expected our respondents to be most likely to overestimate the proportion of immigrants in their neighborhoods (H1). We created an additive index of perceptions of the size of the non-citizen population through combining perceptions of the proportions of documented and undocumented immigrants. As shown in Table 4, when compared to the true size of the non-citizen population from the ACS data, 49 percent of respondents accurately estimated the non-citizen population, while 30 percent underestimated and 20 percent overestimated. Regarding the comparison to the ACS native-born data, around 70 percent of respondents accurately estimated the citizen population, while 22 percent underestimated and 8 percent overestimated the size of the neighborhood's citizen population.

**Table 4:** Accuracy of perceptions of citizen and noncitizen neighborhood populations.

			**95% confidenceinterval**	
**Variable**	***n***	**Sample percentage (%)**	**Lower bound(%)**	**Upper bound (%)**
**CITIZENS**				
Accurate estimation	141	70.2	63.4	76.1
Underestimation	45	22.4	17.1	28.7
Overestimation	15	7.5	4.5	12.1
**COMBINED PERCEPTION: DOCUMENTED AND UNDOCUMENTED IMMIGRANTS**				
Accurate estimation	99	49.3	42.3	56.2
Underestimation	60	29.9	23.9	26.6
Overestimation	42	20.9	15.8	27.1
Number of observations	201			

Thus, respondents were more likely to accurately estimate the proportion of citizens than the proportion of non-citizens, but, notably, they were still more likely to accurately estimate the proportion of non-citizens than to overestimate. In addition, respondents were also much more likely to say that they could not choose in response to the documented and undocumented immigrant questions (7 and 12.5%, respectively) compared to the citizens question (3%). The comparable categories of underestimation of the citizen population and overestimation of the non-citizen population were remarkably consistent, at 22.4 and 20.9%, respectively. Thus, we do not find support for our hypothesis that people would be more likely to overestimate the size of the immigrant population in their respective neighborhood than to underestimate or accurately estimate (H1). In fact, they are most likely to accurately estimate. We also did not expect that so many New Yorkers (30%) would *underestimate* the size of the non-citizen population.

Moreover, although our measures differed, the proportion of misperceptions of non-citizens was lower in our sample than those of similar studies (Alesina et al., 2018; Gorodzeisky and Semyonov, 2019), which lends some support to our second hypothesis that New Yorkers would be less likely (than those in studies of other geographic areas) to overestimate the size of their neighborhoods' non-citizen populations (H2). In particular, previous studies have found that the majority of respondents overestimated the size of the immigrant population (Semyonov et al., 2008; Alesina et al., 2018; Gorodzeisky and Semyonov, 2019). In Alesina et al. (2018) study, the authors find that 86.6% of Americans overestimated the size of the immigrant populations. The numbers are better in some of the other countries they examine, with Italy having the highest proportion of accurate estimators (14.0%, compared to between 5.1 and 10.8% among the other five countries studied) and Sweden having the lowest proportion of overestimators (61.8%, compared to between 70.6 and 86.6% among the other countries studied).^9^ Using similar qualitative categories of misperception, Gorodzeisky and Semyonov (2019) also find that residents of some countries with high proportions of immigrants overestimated the size of the immigrant population. More than 60% of British, French, Belgian, and Dutch citizens overestimated the relative size of the immigrant population in their respective countries.

While we hesitate to make direct comparisons across these studies because of methodological differences in our measure of perceptions (e.g., we asked our respondents to select among qualitative categories arranged along an ordinal scale compared to the two other studies of perceptions of the size of the immigrant population, which asked respondents to estimate the exact numerical percentage), we offer one plausible and intuitive explanation for these differing results. Following Gorodzeisky and Semyonov's (2019) argument, it is possible that our lower proportion of NYC residents misperceiving the size of the non-citizen population may be attributable to New York City's high percentage of foreign-born and non-citizen residents *and* long experience with international migration. Notably, in our study, residents of Queens, the most diverse county in New York and the United States (perhaps the world) were the least likely to overestimate the size of the non-citizen population with only 14% overestimating.

We also estimated a series of logistic regression models to determine if the accuracy of respondents' estimates was related to any demographic characteristics (gender, age, race, level of education, and income). Across models of the proportion of citizens and non-citizens in a respondent's neighborhood, there were consistently no statistically significant effects of any of these characteristics (see Table A2 in Supplementary Material).

## Perceptions and Attitudes

Before we turn to the question of how accuracy of perceptions is related to support for redistribution and social policy, we first examine the association between these attitudes and perceptions themselves (regardless of their accuracy). Our third hypothesis was that those who perceive higher proportions of immigrants in a neighborhood will report lower support for redistribution and social policies in comparison to those who perceive lower proportions (H3).

Below, we examine support for redistribution and social policies among our respondents by perception of the size of the citizen, and documented and undocumented immigrant populations. For mean support for redistribution and social policy along with ANOVA results by level of perceived size of the citizen, and documented and undocumented immigrant populations using the three-category measure of population perceptions, please see the Table A3 in Supplementary Material. In general, support for redistribution and social policy increase as the perceived size of the neighborhood citizen population increases, and decrease as the perceived size of both non-citizen groups increase. However, only the mean differences in support for social policy between perceiving a majority of citizens vs. lower numbers of citizens were statistically significant. These preliminary findings are confirmed through regression analyses.

In Table 5, we summarize the results of bivariate OLS regression models of the effects of the perceived proportion of citizens in a neighborhood on support for redistribution (Model 1) and social policy (Model 3). We also summarize results of multi-variable OLS regression models (Models 2 and 4). We find that the effects of perceived proportion of citizens on support for redistribution are not statistically significant (Models 1 and 2). However, we observe that the effect of the perceived proportion of citizens in a neighborhood has a positive and statistically significant effect on support for social policies (Model 3). This effect remains statistically significant when we control for a range of factors, perceived proportion of white neighbors, the respondent's own race, gender, age, level of education, and household income (Model 4). Thus, those who perceive themselves as living around a higher proportion of citizens are more supportive of social policies.

**Table 5:** OLS regression models of perceptions of size of neighborhood citizen population and support for redistribution and social policy.

**Variables**	**(1)**	**(2)**	**(3)**	**(4)**
	**Redist**	**Redist**	**Soc Pol**	**Soc Pol**
Perception: # of U.S. citizens	0.02 (0.11)	0.10 (0.11)	0.23^**^ (0.08)	0.29^***^ (0.08)
Female		0.17 (0.16)		0.20 (0.12)
Age		−0.01 (0.01)		0.01 (0.01)
Respondent race: White		−0.08 (0.18)		−0.02 (0.13)
College		0.35^*^ (0.16)		0.28^*^ (0.12)
Income		−0.11^**^ (0.04)		−0.11^***^ (0.03)
Perception: # of white neighbors		−0.11 (0.07)		−0.07 (0.05)
Constant	3.49^***^ (0.51)	4.20^***^ (0.56)	1.99^***^ (0.38)	1.93^***^ (0.41)
Observations	201	201	201	201
R-squared	0.00	0.10	0.04	0.15

*Standard errors in parentheses*.

*** *p < 0.001*,

** *p < 0.01*,

* *p < 0.05*.

In Table 6, we summarize the results of bivariate OLS regression models of the effects of the perceived proportion of non-citizen neighbors (Models 1 and 3), along with multi-variable models including controls (Models 2 and 4) on support for redistribution (Models 1 and 2) and social policy (Models 3 and 4). The results demonstrate that the effects of the perception of the size of the non-citizen population in a neighborhood have no statistically significant effects on support for redistribution or social policies with one exception—the near-significance of the negative coefficient of perception of the size of the non-citizen population on support for social policies in the full model (Model 4).

**Table 6:** OLS regression models of perceptions of size of neighborhood non-citizen population and support for redistribution and social policy.

**Variables**	**(1)**	**(2)**	**(3)**	**(4)**
	**Redist**	**Redist**	**Soc Pol**	**Soc Pol**
Perception: # of non-citizens	−0.03 (0.09)	−0.12 (0.10)	−0.10 (0.07)	−0.14^∧^ (0.07)
Female		0.18 (0.16)		0.18 (0.12)
Age		−0.01^∧^ (0.01)		0.01 (0.01)
Respondent race: White		−0.07 (0.18)		−0.04 (0.14)
College		0.38^*^ (0.16)		0.32^**^ (0.12)
Income		−0.11^**^ (0.04)		−0.11^***^ (0.03)
Perception: # of white neighbors		−0.12 (0.07)		−0.05 (0.05)
Constant	3.65^***^ (0.24)	4.95^***^ (0.45)	3.30^***^ (0.18)	3.50^***^ (0.34)
Observations	201	201	201	201
R-squared	0.00	0.10	0.01	0.11

*Standard errors in parentheses*.

*** *p < 0.001*,

** *p < 0.01*,

* *p < 0.05, ^∧^p < 0.1*.

Regarding the control variables included in models in Tables 5 and 6, the coefficients of income are negative and statistically significant in all multi-variable models, and the coefficients of college education are positive and significant. The effects of respondent's race, gender, age, and the perceived proportion of white neighbors are non-significant. The latter suggests that the effect of the perception of the size of the citizen or non-citizen population is not explained by the perception of the size of the white population.

## Misperceptions and Attitudes

We have established that perceptions of the neighborhood citizen population affect support for social policies, but not redistribution. Next, we turn to the question of whether the accuracy of these perceptions is relevant. In particular, we hypothesized that inflated (overestimation) perceptions of the size of the non-citizen population will be associated with lower support for redistribution and social policies compared to accurate estimation or under-estimation (H4).

We begin by examining the effects of the accuracy of perceptions of size of the neighborhood U.S.-citizen population. In Table 7, we summarize the results of bivariate and multi-variable OLS regression models of these effects on support for redistribution (Model 1 and 2) and social policy (Model 3 and 4). The coefficients of underestimation of the neighborhood citizen population (compared to accurate estimation) are negative and statistically significant in the bivariate model of support for social policies (Model 3), as well as the multi-variable model that includes a range of control variables—including perception of the number of white neighbors, race, gender, age, level of education, and household income. However, the effects of overestimation are non-significant in both models of support for redistribution (Models 1 and 2). The coefficients of overestimation of the neighborhood citizen population (compared to accurate estimate) are non-significant in all models.

**Table 7:** OLS Regression Models of Accuracy of Perceptions of Size of Neighborhood Citizen Population and Support for Redistribution and Social Policy.

**Variables**	**(1)**	**(2)**	**(3)**	**(4)**
	**Redist**	**Redist**	**Soc Pol**	**Soc Pol**
**ACCURACY: CITIZENS**^**a**^				
Underestimation	−0.15 (0.19)	−0.30 (0.19)	−0.42^**^ (0.14)	−0.51^***^ (0.14)
Overestimation	−0.25 (0.30)	−0.31 (0.29)	0.02 (0.22)	−0.04 (0.21)
Female		0.17 (0.16)		0.18 (0.12)
Age		−0.01 (0.01)		0.01 (0.01)
Respondent race: White		−0.09 (0.18)		−0.01 (0.13)
College completed		0.36^*^ (0.16)		0.29^*^ (0.12)
Income		−0.12^**^ (0.04)		−0.11^***^ (0.03)
Perception: # of white neighbors		−0.11 (0.07)		−0.07 (0.05)
Constant	3.63^***^ (0.09)	4.79^***^ (0.36)	3.16^***^ (0.07)	3.38^***^ (0.27)
Observations	201	201	201	201
R-squared	0.01	0.11	0.05	0.15

*Standard errors in parentheses*.

*** *p < 0.001*,

** p < 0.01, *p < 0.05.

a *Omitted category is accurate estimate of the neighborhood proportion of citizens*.

In Table 8, we summarize the results of bivariate and multi-variable OLS regression models of the effects of accurate estimation of the proportion of non-citizens in a neighborhood on support for redistribution (Model 1 and 2) and social policy (Model 3 and 4). The coefficients of overestimation of the neighborhood non-citizen population (compared to accurate estimate) are negative and statistically significant in both models of support for social policies, but non-significant in the models of support for redistribution. The coefficients of underestimation of the neighborhood non-citizen population are non-significant in all models.

**Table 8:** OLS regression models of perceptions of size of neighborhood non-citizen population and support for redistribution and social policy.

**Variables**	**(2)**	**(4)**	**(6)**	**(8)**
	**Redist**	**Redist**	**Soc Pol**	**Soc Pol**
**ACCURACY: NON-CITIZENS**^**b**^				
Underestimation	−0.01 (0.18)	0.02 (0.18)	0.00 (0.13)	0.04 (0.13)
Overestimation	−0.05 (0.20)	−0.08 (0.20)	−0.32^*^ (0.15)	−0.33^*^ (0.15)
Female		0.16 (0.16)		0.18 (0.12)
Age		−0.01^∧^ (0.01)		0.01 (0.01)
Respondent race: White		−0.08 (0.18)		−0.03 (0.13)
College completed		0.37^*^ (0.16)		0.32^**^ (0.12)
Income		−0.11^**^ (0.04)		−0.11^***^ (0.03)
Perception: # of white neighbors		−0.09 (0.07)		−0.03 (0.05)
Constant	3.59^***^ (0.11)	4.62^***^ (0.35)	3.13^***^ (0.08)	3.15^***^ (0.26)
Observations	201	201	201	201
R-squared	0.00	0.09	0.03	0.13

*Standard errors in parentheses*.

*** *p < 0.001*,

** *p < 0.01*,

* *p < 0.05*.

b *Omitted category is accurate estimate of the neighborhood proportion of non-citizens*.

Among the control variables in the models in both Tables 7 and 8, consistent with the perceptions models presented in Tables 5 and 6, household income is a negative and statistically significant predictor of both attitudes toward redistribution and social policies, and the coefficients of college education are positive and significant (Models 2 and 4). The coefficients of other control variables are non-significant in all models.

As additional tests of robustness of the social policy attitudes findings above, we examine the following alternative model specifications to compare the results to those in Tables 7 and 8: (1) using OLS with robust-clustered standard errors to control for the lack of independence of observations within neighborhoods; (2) controlling for the actual percentages of citizens and non-citizens in neighborhoods (ACS data); and (3) including a measure of work in high-immigrant industries (excluded from the main models because of missing data). These results are summarized in the Table A4, A5 in Supplementary Material and yield results that are very consistent with those presented above.

## Discussion

In the past several decades, there has been precipitous growth in immigration and a corollary concern regarding the economic and cultural consequences of immigration. The research literatures on ethnic fractionalization, diversity, and racial/ethnic heterogeneity all posit that immigration may undermine social welfare spending and public support for social welfare policies. However, the results of these related literatures provide mixed support for this hypothesis. There appears to be instead, a distinct set of contextual and individual-level characteristics (rising immigration, misperceptions of the size of the immigrant group) that may weaken the public's support for welfare policies. Drawing from a broad set of related studies that argue that people's perceptual realities regarding immigrants and immigration inform attitudes, we examined the extent to which people overestimate the proportion of the immigrant population and its relationship to attitudes about redistribution and social policies. A diverse sample of NYC residents answered a series of questions regarding their perception of the size of the citizen/non-citizen population in their respective neighborhoods of residence and two social policy preferences indicators.

Somewhat in line with our first hypothesis (H1), about a quarter of New Yorkers overestimated the size of the non-citizen population. This overestimation is consistent with other studies, although the proportion of respondents that overestimated was lower, a point we elaborate on more below. Interestingly, more New Yorkers actually gave accurate estimates or underestimated the size of the non-citizen population than overestimated it, lending some support to our second hypothesis that accurate perceptions would be more prevalent in a stable high-immigrant environment (H2). Furthermore, overestimation of the size of the non-citizen population did not differ across our key demographic characteristics (gender, age, race, level of education, household income, employment in high-immigration industry). This finding contrasts with other studies that have found that misperceptions were most extreme among the non-college educated and those working in immigration-intensive sectors (Nadeau et al., 1993; Alesina et al., 2018). However, we emphasize that this is a pilot study with only 201 observations in the analytic sample. The small sample size alone may explain the lack of statistical significance of the demographic traits found to be salient in others' studies.

Our results also suggest some interesting associations between perceptions, accuracy of perceptions, and policy preferences. Somewhat in line with our third hypothesis (H3), those who perceived themselves as living around a higher proportion of citizens were more supportive of social policies, but not redistribution. In contrast, those perceiving higher numbers of non-citizens in their neighborhoods may be less supportive of social policy preferences and redistribution, but these effects were not statistically significant. Together, these results are the first to illustrate that people's perceptions of the size of non-citizen vs. citizen population have differing effects on policy preferences, and that these perceptions are more clearly associated with support for specific social policies than with general attitudes about redistribution.

We were also interested in the question of whether accuracy of these perceptions is germane to policy preferences. We found some support for our final hypothesis (H4) that overestimation of the size of the immigrant population (or underestimation of the size of the citizen population) compared to accurate estimation was associated with lower support for social policies, although neither measure was associated with attitudes toward redistribution. In addition, the large group of respondents who underestimated the non-citizen population did not differ much from respondents who estimated accurately in terms of their support for redistribution or social policies.

## Implications

Taken together, our findings suggest several novel avenues for future research on the formation of public opinion regarding redistributive and social policies. In particular, our research suggests that the majority of our NYC-resident respondents either actually accurately perceived or underestimated the size of the non-citizen population in their respective neighborhoods. While we hesitate to make direct comparisons across these studies because of the methodological differences in our measure of perceptions, we offer one plausible explanation for these differing results. Following Gorodzeisky and Semyonov's (2019) argument, it is possible that the lower proportion of NYC residents misperceiving the size of the non-citizen population may occur because New York City has a high percentage of foreign-born residents *and* has had long experience with international migration; residents of Queens, perhaps the most diverse county in the world, were the least likely to overestimate the size of the non-citizen population. Moreover, unlike other high-immigrant population countries like France and Belgium, New York City has historically advanced a diverse and multicultural ideology premised on inclusion and the value of different groups. It is possible that through an emphasis of these values and the city's lengthy history of high rates of immigration, NYC residents may have more opportunities to acquire a particular type of knowledge regarding the size of the immigrant population, similar to the Swiss who were the most accurate estimators in the Gorodzeisky and Semyonov (2019) study. This is also consistent with Alesina et al. (2018) finding that respondents who knew an immigrant personally had more accurate perceptions. An additional interpretation for a relatively low rate of overestimation of the size of the immigrant population in our sample could result from a ceiling effect. In particular, it is possible that once the share of an immigrant population is very high, as it is in New York City, there may be less of a chance of overestimation. Future research should further explore how the role of perceptions of immigrants differ across new vs. traditional immigrant-receiving contexts.

Second, to our knowledge, our study is one of the few to examine and illustrate that policy preferences may be in part a function of people's perceptions regarding the size of the immigrant population. This is consistent with a related set of studies that have shown that misperceptions are associated with anti-immigrant attitudes (Semyonov et al., 2004, 2008; Herda, 2013; Pottie-Sherman and Wilkes, 2017; Gorodzeisky and Semyonov, 2019). Our results also provide some preliminary evidence that misperceptions (e.g., overestimating the size of the immigrant population) undermine public support for social welfare policies, but only among one of our two attitudinal indices. In interpreting these findings, it is possible that the two indices are capturing different components of social welfare attitudes. For example, the redistribution questions (Index 1) are broader and theoretical and may be tapping into respondents' ideologies related to social welfare. The social policy questions (Index 2), in contrast, are more specific and ask respondents to consider the application of targeted social welfare policies. This may mean that respondents' perceptions of the size of the non-citizen population are unrelated to the “principles” of social welfare, but negatively related to the application of those principles.

Finally, unlike previous studies, we measure perceptions of the size of the majority population—U.S. citizens—along with the size of the minority immigrant populations. Furthermore, to the best of our knowledge, we are also the first researchers to ask about perceptions of the size of the documented vs. undocumented immigrant populations. Although we did not necessarily expect respondents to be able to distinguish between the two groups with a great deal of accuracy (Flores and Schachter, 2018), we expected perceptions of the size of the two types of immigrant groups to be meaningful. However, we do not find any statistically significant differences between perceptions of groups of different legal immigration statuses on preferences for redistribution or social policy. Differences may emerge in studies with larger sample sizes. On the other hand, our data suggest that perceptions of proportions of citizens compared to proportions of non-citizens in neighborhoods may not have identically inverse implications for attitudes about social policies, with effects potentially being more pronounced when respondents considered the size of the citizen population. While we are, again, limited by the fact that this was a pilot study with a relatively small number of observations, we believe that the evidence about citizen perceptions suggests an important avenue for future research.

In considering the results of the study, it is important to note some limitations, some of which present opportunities for future research. The most important caveat is that respondents are not randomly assigned to neighborhoods of residence, an option that is rarely available and ethically fraught. To the extent that respondents choose where they live, these choices may reflect broader values and ideologies, which might drive the associations observed. In addition, the data we analyzed were cross-sectional, which prevents us from evaluating the causal or directional order of our main theoretical variables. In addition, our measure of respondents' perceived size of the immigrant group was ordinal in nature and did not exactly align with the ACS categories of the specific numbers of citizen and non-citizen populations. Thus, future research might ideally employ more comprehensive quantitative measures of the perceived immigrant population similar to Alesina et al. (2018). One final issue that is that we did not collect data that would have allowed us to determine whether the perceived characteristics of the non-citizen population are related to policy preferences. Alesina et al. (2018), for example, found that not only did respondents have strongly misinformed views about the size of the immigrant population in general, they also overestimated the share of immigrants from non-Western and Muslim majority countries while underestimating the share of Christian migrants. These misperceptions, in turn, made natives more opposed to redistribution, and were more salient predictors than estimations of the size of the immigrant population as a whole. Future research should continue to unpack the characteristics of the immigrant population as it relates to attitudes toward social policies.

While we do not want to draw any firm conclusions from a small pilot study, this study offers important insights into how the perceived size of the non-citizen population may affect social policy attitudes. In particular, our results suggest that a subset of respondents overestimate the size of the non-citizen population. These misinformed individuals are also the least supportive of social welfare policies. We hope that future research will further examine source misrepresentations about immigrants and the related implications for public opinion.

## Data Availability

The datasets generated for this study are available on request to the corresponding author.

## Author Contributions

LS and KP: Conceptualization, Investigation, Writing—Original Draft, Writing—Review and Editing; LS: Data curation, formal analysis, funding acquisition, methodology, visualization.

### Conflict of Interest Statement

The authors declare that the research was conducted in the absence of any commercial or financial relationships that could be construed as a potential conflict of interest.

## Footnotes

^1^The requirements were that participants have an HIT approval rate greater than or equal to 95%, and that the number of HITs completed be greater than or equal to 1,000.

^2^Men, younger people, political liberals, those who are less religious, and white people tend to be over-represented in mTurk samples compared to the U.S. population (Berinsky et al., 2012).

^3^For example, inconsistencies between zip code and neighborhood name (an open-ended question); providing a non-existent neighborhood name; or discrepancies between the name of the neighborhood and/or zip code and the borough selected.

^4^We treated the questions from these two surveys as separate constructs after an examination of orthogonally rotated factor loadings.

^5^There is a substantial debate about whether midpoint categories, such as the “neither agree nor disagree” response option for the 2009 questions, should be treated as true middle categories or as equivalent to non-response, but no clear consensus (Krosnick and Presser, 2010). We contend that a midpoint response is highly valid in the context of policy preferences, and thus do not exclude these respondents from our analyses.

^6^These figures include 24 respondents (7.5%) who selected more than one racial or ethnic category.

^7^Although the measures are ordinal, they are frequently treated as continuous in the literature. Moreover, ordinal logistic regression models yield nearly identical results in terms of the directionality and statistical significance of the results presented in this paper, although the interpretation would be much less accessible to many readers.

^8^We considered structural equation modeling (SEM) as an alternative. To quantify the potential value of SEM in our case, we compared standardized coefficients of models with our additive index outcomes to those predicted after factor analysis (in which the ranges of the factor loadings−0.75 to 0.82 in the case of Index 1, and 0.79 to 0.84 in the case of Index 2—were very narrow). Given that the standardized coefficients in both sets of models were virtually identical, we do not believe that the added analytical complexity of SEM compared to OLS is justified.

^9^Because respondents in the Alesina et al. (2018) study were asked to provide an exact percentage of immigrants in the country, and were only classified as accurate if they were within two percentage points of the true value, it is less surprising that so many respondents were inaccurate. However, if there were no bias in these estimations, we would expect respondents to over- and underestimate at the same rates, and that is not the case.
