# Supplementary material for: The Effects of Perceived Neighborhood Immigrant Population Size on Preferences for Redistribution in New York City: A Pilot Study
Source: Front Sociol. 2019 Mar 26;4:18. doi: 10.3389/fsoc.2019.00018 (PMC8022475; doi:10.3389/fsoc.2019.00018)
Supplement: Supplementary file 1 [file Data_Sheet_1.pdf]

Appendix

Table A1. Construction of Measure of Actual vs. Perceived Neighborhood Foreign-Born Population

Actual ACS percentage	Perception category	Classification
Citizen (range: 37-85%)		
37-39.9%	• Less than half	Accurate
	• None, almost none, very few	Underestimate
	• Close to half, more than half	Overestimate
40-49.9%	• Close to half	Accurate
	• None, almost none, very few, less than half	Underestimate
	• More than half	Overestimate
>=50%	• More than half	Accurate
	• [all others]	Underestimate
Non-citizen (range: 3.5%-35.7%)		
3.5-10%	• Almost none, very few	Accurate
	• None	Underestimate
	• [all others]	Overestimate
10-29.9%	• Less than half	Accurate
	• None, almost none, very few	Underestimate
	• Close to half, more than half	Overestimate
>=30%	• Less than half, close to half	Accurate
	• None, almost none, very few	Underestimate
	• More than half	Overestimate

Table A2. Logistic Regression Models of Effects of Demographic Characteristics on Accuracy of Perceptions of Proportion Citizen and Non-Citizen Neighbors

	(1)	(2)	(3)	(4)	(5)	(6)
	Citizens			Non-citizens		
Estimation:	Accurate	Under	Over	Accurate	Under	Over
Female	-0.18	0.43	-0.59	0.16	-0.57	0.44
	(0.33)	(0.36)	(0.62)	(0.30)	(0.34)	(0.37)
Age	0.00	-0.01	0.02	0.02	-0.01	-0.01
	(0.02)	(0.02)	(0.03)	(0.02)	(0.02)	(0.02)
White	0.39	-0.10	-0.89	-0.25	0.13	0.22
	(0.34)	(0.37)	(0.56)	(0.32)	(0.36)	(0.40)
College	0.14	-0.37	0.56	-0.22	0.04	0.29
completed	(0.34)	(0.36)	(0.64)	(0.31)	(0.34)	(0.39)
Income	0.09	-0.07	-0.12	0.01	-0.04	0.03
	(0.09)	(0.10)	(0.17)	(0.08)	(0.09)	(0.09)
Constant	0.11	-0.47	-2.35*	-0.33	-0.32	-1.58*
	(0.64)	(0.71)	(1.13)	(0.59)	(0.66)	(0.75)
Observations	201	201	201	201	201	201
Pseudo R-squared	0.02	0.02	0.04	0.01	0.02	0.01

Standard errors in parentheses

* p<0.05

Table A3. Analysis of Variance (ANOVA) results of support for redistribution and social policies by perceptions of the neighborhood citizen and non-citizen populations.

		Index 1: Redistribution Mean (SD)	Index 2: Social Policies Mean (SD)
**Citizens**	*None/very few* *n = 3*	3.78 (0.69)	1.83 (0.52)
	Less than/close to half *n = 52*	3.51 (1.03)	2.90 (0.85)
	More than half *n = 146*	3.59 (1.12)	3.15 (0.80)
	ANOVA	$F(2,199)=.63$, $p=.535$	$F(2,198)=5.36$, $p=.005$[1]
**Documented Immigrants**	*None/very few* *n = 66*	3.70 (1.10)	3.22 (0.69)
	Less than/close to half *n = 124*	3.54 (1.08)	3.00 (0.88)
	More than half *n = 11*	3.18 (1.04)	2.89 (0.97)
	ANOVA	$F(2,198)=1.22$, $p=.298$	$F(2,198)=1.85$, $p=.160$
**Undocumented Immigrants**	*None/very few* *n = 149*	3.60 (1.13)	3.10 (0.83)
	Less than/close to half *n = 51*	3.52 (0.99)	3.01 (0.84)
	More than half *n = 1*	3.33 (0)	1.75 (0)
	ANOVA	$F(2,198)=.13$, $p=.880$	$F(2,198)=1.47$, $p=.232$

[1] A Tukey post hoc test revealed that those who believed more than half of their neighbors were citizens were statistically significantly more supportive of social policies (.82 ± .32, p = .027) than those who believed very few or none of their neighbors were citizens, and more supportive of social policies (.35 ± .11, p = .004) than those who believed that less than or close to half of their neighbors were citizens. There was no statistically significant difference between the very few/none category and the less than/close to half category (p = .313).

Table A4. Analysis of Variance (ANOVA) results of support for redistribution and social policies by accuracy of perceptions of the neighborhood citizen and non-citizen populations

		**Index 1: Redistribution Mean (SD)**	**Index 2: Social Policies Mean (SD)**
**Citizens**	*Accurate* *n = 141*	3.63 (1.11)	3.16 (0.80)
	Overestimate *n = 15*	3.38 (1.23)	3.18 (0.77)
	Underestimate *n = 45*	3.47 (1.00)	2.74 (0.88)
	ANOVA	*F*(2,198)=.61, *p*=.546	*F*(2,198)=4.70, *p*=.010[2]
**Non-citizens**	*Accurate* *n = 99*	3.59 (1.09)	3.13 (0.80)
	Overestimate *n = 42*	3.54 (1.00)	2.81 (0.98)
	Underestimate *n = 60*	3.58 (1.16)	3.14 (0.73)
	ANOVA	*F*(2,199)=.04, *p*=.965	*F*(2,199)=2.60, *p*=.077[3]

[2] A Tukey post hoc test revealed that those who accurately estimated (.45 ± .11, p = .000) and those who underestimated (.51 ± .19, p = .022) the size of citizen population were statistically significantly more supportive of social policies than those who overerestimated. There was no statistically significant difference between the accurate and over-estimators (p = .939).

[3] A Tukey post hoc test revealed that those who overestimated the size of the neighborhood non-citizen population (-.32 ± .13, p = .038) were statistically significantly more supportive of social policies than those who underestimated. There were no statistically significant differences between the accurate and under-estimators (p = .963), nor between over- and under-estimators (p=.106).

Table A5. Alternative OLS Regression Models of Perceptions of Size of Neighborhood Citizen Population and Support for Social Policy

	(1)†	(2)	(3)
VARIABLES	Soc Pol	Soc Pol	Soc Pol
Neighborhood-level			
% Citizen		-0.75	
Population		(0.58)	
Individual-level			
Accuracy: Citizens[A]			
Underestimation	-0.51**	-0.53***	-0.58***
	(0.15)	(0.14)	(0.15)
Overestimation	-0.04	-0.22	-0.12
	(0.21)	(0.26)	(0.24)
Female	0.18	0.18	0.18
	(0.14)	(0.12)	(0.13)
Age	0.01	0.01	0.01
	(0.01)	(0.01)	(0.01)
Respondent race:	-0.01	-0.01	-0.00
White	(0.14)	(0.13)	(0.14)
College completed	0.29*	0.28*	0.28*
	(0.13)	(0.12)	(0.13)
Income	-0.11***	-0.11***	-0.11**
	(0.02)	(0.03)	(0.03)
Perception: # of	-0.07	-0.05	-0.05
white neighbors	(0.05)	(0.06)	(0.06)
Employed in			-0.05
high-immigrant industry			(0.12)
Constant	3.38***	3.81***	3.36***
	(0.26)	(0.43)	(0.30)
Observations	201	201	177
R-squared	0.15	0.16	0.17
Clusters	51		

Standard errors in parentheses
*** p<0.001, ** p<0.01, * p<0.05
† With Robust-Clustered Standard Errors
[A] Omitted category is accurate estimate of the neighborhood proportion of citizens

Table A6. Alternative OLS Regression Models of Perceptions of Size of Neighborhood Non-Citizen Population and Support for Social Policy

	(1)	(2)	(3)
VARIABLES	Soc Pol	Soc Pol	Soc Pol
Neighborhood-level			
% Non-citizen		0.11	
Population		(1.04)	
Individual-level			
Accuracy: Non-citizens[B]			
Underestimation	0.04	0.04	0.00
	(0.12)	(0.13)	(0.15)
Overestimation	-0.33*	-0.33*	-0.36*
	(0.15)	(0.15)	(0.16)
Female	0.18	0.18	0.16
	(0.14)	(0.12)	(0.13)
Age	0.01	0.01	0.01
	(0.01)	(0.01)	(0.01)
Respondent race:	-0.03	-0.03	0.01
White	(0.15)	(0.14)	(0.15)
College completed	0.32*	0.32**	0.34*
	(0.13)	(0.12)	(0.13)
Income	-0.11***	-0.11***	-0.11**
	(0.02)	(0.03)	(0.03)
Perception: # of	-0.03	-0.02	-0.01
white neighbors	(0.05)	(0.06)	(0.06)
Employed in			-0.09
high-immigrant industry			(0.13)
Constant	3.15***	3.12***	3.12***
	(0.25)	(0.34)	(0.30)
Observations	201	201	177
R-squared	0.13	0.13	0.13
Clusters	51		

Standard errors in parentheses
*** p<0.001, ** p<0.01, * p<0.05

[†] With Robust-Clustered Standard Errors
[B] Omitted category is accurate estimate of the neighborhood proportion of non-citizens
